# Comparison Between Effect of Letrozole Plus Misoprostol and Misoprostol Alone in Terminating Non-Viable First Trimester Pregnancies: A Single Blind Randomized Trial 

**Published:** 2018-03

**Authors:** Fatemeh Abbasalizadeh, Farnaz Sahhaf, Paria Sadeghi-Shabestari, Mohammad Mirza-Aghazadeh-Attari, Mohammad Naghavi-Behzad

**Affiliations:** 1 Department of Gynecology and Obstetrics, Women’s Reproductive Health Research Center, Tabriz University of Medical Sciences, Tabriz, Iran; 2 Medical Philosophy and History Research Center, Tabriz University of Medical Sciences, Tabriz, Iran ; Students’ Research Committee, Tabriz University of Medical Sciences, Tabriz, Iran; 3 Students’ Research Committee, Tabriz University of Medical Sciences, Tabriz, Iran

**Keywords:** Letrozole, Misoprostol, Medical Abortion, First Trimester, Pregnancy

## Abstract

**Objective:** To evaluate the effect of letrozole plus misoprostol to terminate non-viable pregnancies in first trimester compared with the use of misoprostol alone.

**Materials and methods:** In a single-blind clinical trial, 128 women over 18 years old referred to Educational-Medical centers of Tabriz University of Medical Science (Tabriz, Iran), for abortion in first trimester of non-viable pregnancies, were randomly selected in two intervention and control groups using Rand list (version 1.2) software. To complete abortion both groups received 600 mcg of misoprostolorally. The intervention group received letrozole 10 mg daily for 3 days before receiving misoprostolorally. Complete abortion rate and the side effects of both groups were recorded.

**Results:** Mean pregnancy age based on LMP in intervention group and control group were 7.74 ± 0.95 and 8.52 ± 1.29 weeks respectively. Complete abortion rate in the intervention group was 93.7%, and in control group was 68.7% which was significantly higher in intervention group (p = 0.001). Abdominal pain in the intervention group is also significantly lower than that of the control group (p = 0.013). Intervention group also had significantly lower duration of bleeding rather than control group (p = 0.006).

**Conclusion:** Based on the findings of this study, letrozole pretreatment with misoprostol for first-trimester medical abortion can increase complete abortion rate significantly without increasing side effects compared to use of misoprostol alone.

## Introduction

Based on the reports of WHO, 53 million abortion cases occur annually (1, 2). Inducing abortion by drugs is vastly used in global level and its use has increased since 1950 (3, 4). Selected method for terminating pregnancy in 1960s was vacuum aspiration surgery, and then by manufacturing mifepristone in 1980s, usage of pregnancy termination methods by drugs increased (5).

Inducing abortion by drugs is an alternative for surgery intervention, which has economic benefits with lower side effects and its rate of success is 60 to 95 percent (6, 7). In medical therapy method various drugs could be used to induce abortion. Considering limitation in access to mifepristone drugs and its high cost, it is not accessible in most of the countries and alternative drugs are used to induce abortion (8). One of these drugs which used both vaginally and orally is prostaglandin E1 analogue, misoprostol, which is known as Cytotec trade mark. Misoprostol is a cheap drug which could be kept in room temperature and usually used as vaginally and orally (9).

Besides being affordable and efficient, misoprostol has lower side effects and doesn’t need special care during use. This drug is well tolerated by patients and reduces treatment costs significantly and also significantly reduces curettage and need for surgical intervention (10).

Letrozole is also one of aromatase inhibitors, which is used to stimulate ovulation in infertile women suffering ovulatory dysfunction. This drug is one of the main drugs for inhibiting aromatase, with a relatively short 45-hour half life, which is active orally and inhibits aromatase enzyme reversibly. By this drug, estrogen synthesis block leads to increase endogenous gonadotropin and finally stimulates growth of ovarian follicles, and also this drug could play a role in abortion therapy via inhibiting estrogen synthesis (11, 12). This drug is also used to breast cancer related to estrogen (13).

Some studies suggest prescription of aromatase inhibitors prior to use of main drug such as misoprostol or mifepristone for inducing drug abortion, increases efficiency of treatment regimen and alsodecreases the need for surgical interventions (14, 15). Today, pregnancy termination methods in first trimester are widespread but most of these methods are not available in most of the countries. Therefore identifying and diagnosing the best available regimen for drug abortion is very important. Some conducted studies have mentioned to reinforcing the impact of misoprostol with letrozole (14), but reaching firm results need further studies.

Misoprostol is a relatively safe drug and of its side effects it is possible to mention to gestational effects, feeling of warmth, and shivering. Severe side effects of misoprostol consumption are severe bleeding and endometritis, with less than 1% prevalence based on studies (4). Short time use of letrozole in drug abortion had no severe consequences and its usual side effects are sweating, arthralgia, and fatigue, but could decrease bleeding resulted from medical abortion (12, 15).

Abortion in the first trimester of pregnancy with a live fetus except in specific cases is not legal in our country, so present study was conducted on non-viable pregnancies. The objective of conducting this study was to compare effect of misoprostol with letrozole and misoprostol alone in terminating non-viable pregnancies in the first trimester of pregnancy.

## Materials and methods

In a single-blind randomized clinical trial with IRCT code of IRCT2014030114293N1, 128 women over 18 years old who referred to Medical-Educational centers of Tabriz University of Medical Science (Tabriz, Iran) with non-viable under 12-week pregnancy, based on Last Menopause Period (LMP), during June 2013 to April 2015, were randomly selected using Rand list (version 12) software and were Investigated as two groups of intervention and control. 

Inclusion criteria were consisted of minimum age of 18 years for mother, first trimester of pregnancy (less than 12 weeks based on LMP), non-viable fetus (missed abortion or blighted ovum), lack of any maternal diseases such as: heart disease, asthma, history of thromboembolism, cancer, renal failure, and liver diseases, and consent of patient and her spouse to participate in the study. Also exclusion criteria were as followed: any medical problem in patient which need to interfere and emergency treatment, or history of allergy to misoprostol or letrozole drugs.

**Figure 1:**
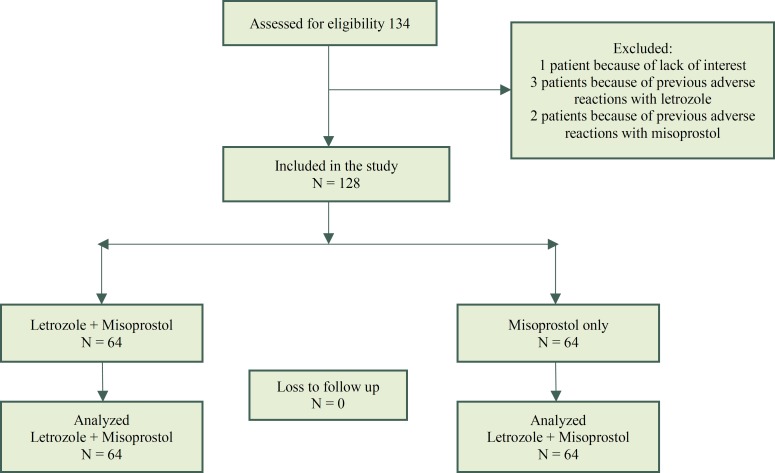
Consort diagram of the study

Sixty-four patients in intervention group as well as 64 patients in control group were included into the study. The consort diagram is included in figure 1. In intervention group to induce drug abortion, patients first daily received 10 mg oral letrozole for 3 days, then they received 600 microgram single dose oral misoprostol, and in control group patients first daily received placebo of letrozole like intervention group and then 600 microgram single dose oral misoprostol was used. Patients in both groups, who had spontaneous abortion before taking misoprostol in the first three days of study, were excluded from the study. For patients in both groups hemoglobin levels were asked at the beginning and end of the study. Serum estradiol levels were measured at the beginning in control group, and also were measured at the beginning and before taking misoprostol in intervention group. Through the study single blinding was applied and patients were not aware of studied groups (Figure 1).

Both groups were monitored for 4 hours after receiving single dose of misoprostol and were examined for possible side effects such as abdominal cramp or possible bleeding, and in terms of lack of abdominal cramp or severe bleeding, were released after explaining risk and warning signs such as bleeding more than normal menstruation. Next visit and monitoring of patients in both groups was conducted 15 days later and response to treatment and complete abortion, amount and duration of bleeding, drug side effects such as nausea, vomiting, diarrhea, headache, dizziness, pain in the lower parts of the abdomen, and ague all were examined and patients in both groups were asked for ultrasound to check incomplete abortion and measuring the hemoglobin level. In case of failure in full disposal of the remnants of pregnancy or failure in termination of pregnancy, repeated doses of misoprostol or curettage candidates underwent surgery and patients in both groups of control and intervention were put in two categories of response to treatment and failure in response to treatment.

In this study patients were included into the study after acquiring informed written consent and also the study protocol was approved by the Ethics Committee of Tabriz University of Medical Sciences (TUMS), which was in compliance with Helsinki Declaration.

Statistical analysis was performed by SPSS software package version 16.0 for windows (SPSS Inc., Chicago, USA). Quantitative data were presented as mean ± standard deviation (SD), while qualitative data were demonstrated as frequency and percent (%).To compare qualitative variables chi square statistical test was used and to compare quantitative variables between two studied groups independent T-test was implemented. Also to compare intra-group quantitative variables such as hemoglobin, paired T-test was applied. In all studied cases, significance level was considered as p < 0.05.

## Results

Mean age of patients in intervention group was 29.21 ± 4.08 years and that of control group was 28.53 ±5.24 years (p = 0.402). Mean pregnancy age based on LMP was 7.74 ± 0.95 weeks in intervention group and 8.52 ± 1.29 weeks in control group. Also mean pregnancy age based on ultrasound was 8.09 ± 0.90 weeks in intervention group and 8.80 ± 1.17 weeks in control group. Two under studied groups had no significant difference in terms of pregnancy age (p = 0.103).

In intervention group 17.2% of mothers had referred because of blighted ovum and 82.8% of them had referred for missed abortion and these ratios in control group were 14.1% and 85.9% respectively.

**Table 1:** Hemoglobin level (mg/dl) in both Intervention and Control groups

	**Intervention group** **Letrozole + Misoprostol**	**Control group** **Placebo + Misoprostol**	**p value**
Before Intervention	13.29 ± 0.95	13.02 ± 0.86	0.080
After Intervention	12.55 ± 1.01	12.14 ± 0.97	0.015

In terms of response to treatment and complete abortion in intervention group 93.75% of women had abortion after receiving letrozole and misoprostol, and only 4 persons (6.25%) didn’t have complete abortion and were candidates for surgical curettage. In control group, on the other hand, 68.75% of people had abortion after receiving misoprostol and 31.25% of them didn’t have full abortion and were candidate for other treatment. In terms of response to treatment two groups had statistically significant difference (p = 0.001).

In intervention group only 4 (6.25%) of patients were candidates for surgical curettage, and in control group 12 patients (18.75%) underwent surgical curettage. Therefore need for surgical curettage in intervention group was significantly lower than that of control group (p = 0.012).

Hemoglobin levels of both studied groups were based on Table 1. Although both intervention and control groups had lower hemoglobin level at the end of the study comparing with that in beginning of the study, but decrease of hemoglobin in the intervention group was statistically significantly lower than control group (p = 0.001).

Median serum estradiol level at beginning if study in control group was 431.05 (pg/ml) with range of 15.70 to 1710.4 (pg/ml), and median serum estradiol level before taking letrozole in intervention group was 442.20 (pg/ml) with range of 11.90 to 1781.0 (pg/ml). At beginning of the study, serum estradiol levels of both groups had no significant difference (p = 0.756). Also, median serum estradiol level after taking letrozole and before taking misoprostol in intervention group was 40.4 (pg/ml) with range of 4.5 to 176.4 (pg/ml). Decrease in serum estradiol level after taking letrozole in intervention group was statistically significant (p = 0.001).

Drugs side effects in two studied groups during the study have been presented in Table 2 which abdominal pain in intervention group was significantly lower than that of control group (p = 0.013).

Generally comparing both groups in terms of duration of bleeding, intervention group had significantly lower duration of bleeding rather than control group (p = 0.006) (Table 3).

## Discussion

Medical abortion decreases side effects such as bleeding and infection, and also stress in patients, comparing with surgery. In medical abortion method it is possible to use various drug regimens to induce abortion. Misoprostol is a prostaglandin analog and a safe and cheap drug which is used for inducing drug abortion and numerous studies have shown its efficiency (16, 17).

In a pilot study, in inducing abortion in 40 legal under-63-day abortion cases, 7.5 mg letrozole for 2 days, and then usage of vaginal misoprostol leads to induce abortion in 80% of patients and comparing with Mifepristone, combination of letrozole with misoprostol was more efficient than combination of letrozole and mifepristone (18). In the current study also success rate of complete abortion was higher in using misoprostol and letrozole comparing with using misoprostol alone (p = 0.001).

**Table 2:** Complications during study in both Intervention and Control groups

	**Intervention group** **Letrozole + Misoprostol**	**Control group** **Placebo + Misoprostol**	**p value**
Nausea	59.3%	70.3%	0.218
Vomiting	29.6%	26.6%	0.710
Diarrhea	31.2%	43.8%	0.113
Dizziness	28.1%	25.0%	0.848
Headache	6.2%	4.7%	0.728
Abdominal pain	54.6%	76.6%	0.013
Fever	6.2%	1.6%	0.127
Chills	10.9%	9.4%	0.786

**Table 3:** Bleeding durationin both Intervention and Control groups

	**Intervention group** **Letrozole + Misoprostol**	**Control group** **Placebo + Misoprostol**	**p value**
Within a few hours	0.00%	7.81%	p = 0.001
Within a day	60.93%	42.19%	p = 0.001
More than a day	39.06%	50.00%	p = 0.001

In a prospective study success rate in inducing abortion for 56-day or less pregnancies using single dose of intravaginal misoprostol, was reported 72%. Also this study claimed that in case of failure in access to mifepristone, using misoprostol alone clinically could lead to acceptable results (19). Also a review study has shown that in treatment of missed abortion in the first trimester of pregnancy by misoprostol, 800 microgram intravaginal misoprostol as single dose or 600 microgram single dose sublingual misoprostol are proper alternatives for surgery. This study also expressed that after prescribing misoprostol there was no need for hospitalization and patients should refer in case of severe bleeding or infection and second investigation and monitoring of patients after 1 to 2 weeks after prescribing misoprostol is suggested (20). In the current study also induction rate of complete misoprostol for using misoprostol alone was 68.75%, and in case of lack of access to another drug, using this drug had acceptable results like other studies.

In a pilot study, up to the ninth week of pregnancy in 50 person, 200 mg single dose mifepristone and daily 10 mg letrozole for 3 days, and finally 800 microgram single dose intravaginal misoprostol, were used to induce abortion rate of complete abortion in this study was 98%, and also no important side effects were reported (21).

Like recent study, in a clinical trial, on 168 women with under-63-day pregnancy age, letrozole therapy before misoprostol was investigated. Success rate in complete abortion in letrozolereceiving group was significantly higher than that of group receiving misoprostol alone. Among side effects, vomiting in letrozole group was significantly higher than control group (22). In recent study using letrozole did not lead to increase in side effects and even abdominal pain and bleeding time in intervention group was significantly lower than that of control group. Also another study showed that using letrozole for 7 days prior to use of vaginal misoprostol increases rate of complete abortion up to 96% (14).

About effect of using letrozole with misoprostol for inducing abortion except the first trimester of pregnancy, in a clinical trial on effect of using letrozole with misoprostol for inducing abortion in the second trimester of pregnancy, prescribing letrozole prior to misoprostol did not lead to significant difference in success rate of inducing medical abortion in the second trimester of pregnancy. Also in this study there was not significant difference between two groups in terms of side effects (23). Similar to the recent study, numerous studies have declared effectiveness of using letrozole prior to misoprostol in increasing rate of success in complete drug abortion, without increase in side effects (24, 25). Also in current study, while comparing bleeding time, letrozole receiving group had mean lower bleeding time and about hemoglobin level, two groups didn’t have significant difference before intervention, but after intervention and conducting abortion, hemoglobin rate of letrozole receiving group was significantly higher than that of control group. Also abdominal pain in letrozole receiving group was lower than that of control group. Finally numerous new studies express that, implementing effective drug regimens for inducing abortion is more affordable and risk free than methods such as vacuum aspiration (26, 27), which should be paid attention by physicians.

## Conclusion

In this study, prescribing letrozole prior to misoprostol was effective in increasing efficiency of misoprostol for inducing complete abortion of non-viable fetus in the first trimester of pregnancy, and also prescribing letrozole didn’t cause side effects in patients and also abdominal pain and time of bleeding in patients was significantly lower.

Using letrozole prior to misoprostol could increase success rate of inducing complete abortion by misoprostol in the first trimester of pregnancy, without increasing side effects.
